# Systemic Molecular Network Alterations Associated with FoxA2 in Gastric Cancer

**DOI:** 10.3390/ijms27146126

**Published:** 2026-07-09

**Authors:** Nurcan Umur, Funda Kosova, Bahadır Çetin, Özgü Kemal Beksaç, Furkan Sağdıç

**Affiliations:** 1 Department of Molecular Biology, Vocational School of Health Services, Manisa Celal Bayar University, Manisa 45030, Turkey; 2 Department of Medical Biochemistry, Vocational School of Health Services, Manisa Celal Bayar University, Manisa 45030, Turkey; fundakosova@gmail.com; 3 Department of General Surgery, Ankara Oncology Training and Research Hospital, Health Sciences University, Ankara 06290, Turkey

**Keywords:** gastric cancer, FOXA2, Ang-1, ApoE4, PEN-2, NF-κB, angiogenesis, inflammation

## Abstract

Gastric cancer is a multifactorial disease characterized by complex interactions among transcriptional regulation, inflammation, metabolic adaptation, and angiogenesis. However, systemic molecular relationships linking these processes remain insufficiently understood. This study aimed to evaluate circulating levels of FOXA2 (Forkhead Box A2), Ang-1 (Angiopoietin-1), ApoE4 (Apolipoprotein E4), PEN-2 (Presenilin Enhancer-2), and NF-κB (Nuclear Factor kappa B) in gastric cancer patients and to explore their potential integrated role in disease biology. Serum samples were obtained from 40 patients with gastric cancer (20 preoperative and 20 postoperative) and 20 healthy controls. Protein levels were measured using enzyme-linked immunosorbent assay (ELISA), followed by statistical analysis. Serum levels of FOXA2, ApoE4, PEN-2, and NF-κB were significantly decreased in gastric cancer patients compared with healthy controls, whereas Ang-1 levels were significantly increased. No statistically significant differences were observed between preoperative and postoperative groups. These findings indicate coordinated dysregulation of transcriptional, inflammatory, metabolic, and angiogenic processes in gastric cancer. The identified FOXA2–NF-κB–PEN-2–ApoE4–Ang-1 axis may be considered an integrated circulating molecular profile reflecting tumor host interactions and may provide a potential foundation for future non-invasive biomarker development and translational research. This study proposes systems-level circulating molecular network model rather than isolated biomarker alterations.

## 1. Introduction

Gastric cancer is one of the leading causes of cancer-related mortality worldwide, ranking second among men and third among women [1]. Its development is influenced by genetic, epigenetic, environmental, and infectious factors, including Helicobacter pylori infection, chronic gastritis, excessive consumption of salty and smoked foods, smoking, alcohol use, and insufficient intake of fruits and vegetables [2].

The molecular biology of gastric cancer is highly complex and involves interactions among transcription factors, inflammatory signaling pathways, lipid-associated proteins, membrane protease complexes, and angiogenic regulators that collectively govern tumor initiation, progression, metastasis, and therapeutic resistance [3]. Understanding these interconnected mechanisms is essential for identifying novel diagnostic biomarkers and targeted therapeutic strategies. Molecules such as Forkhead Box proteins (Fox) A2, angiopoietin (Ang)-1, Apolipoprotein E4 (ApoE4), Presenilin Enhancer-2 (PEN-2), and Nuclear factor kappa B (NF-κB) have emerged as important regulators of gastric cancer cell behavior and tumor microenvironment interactions [4].

Fox proteins constitute a large family of transcription factors characterized by a conserved DNA-binding “forkhead” domain involved in regulating cell growth, differentiation, metabolism, apoptosis, and immune responses [5]. The FoxA subfamily (FoxA1–3) plays essential roles in gastrointestinal organ development by promoting chromatin accessibility and transcriptional activation [6]. Increasing evidence indicates that Fox proteins contribute to cancer progression by modulating proliferation, angiogenesis, metastasis, and drug resistance. FOXA2, in particular, has been reported to regulate gene expression in gastric, lung, and liver cancers and may function either as a tumor suppressor or promoter depending on cellular context [7,8]. Therefore, elucidating the functions of Fox proteins is of great importance for understanding cancer biology and identifying novel therapeutic targets.

NF-κB is a key transcription factor regulating inflammation, immune responses, proliferation, and apoptosis [9]. Normally inactive in the cytoplasm, NF-κB becomes activated in response to cytokines, oxidative stress, and bacterial components, translocating to the nucleus to regulate target gene expression. Constitutive activation of NF-κB has been observed in multiple malignancies, including gastric cancer, where H. Pylori-induced signaling promotes chronic inflammation and carcinogenesis [10,11].

The Ang family regulates vascular maturation and stability through activation of the Tie-2 receptor and downstream PI3K/Akt signaling [12]. Ang-1 maintains vascular integrity and suppresses inflammation under physiological conditions, whereas dysregulation of the Ang–Tie2 axis contributes to abnormal angiogenesis and tumor progression [13].

PEN-2, a membrane protein localized in the endoplasmic reticulum and Golgi apparatus, is an essential component of the γ-secretase complex responsible for intramembrane proteolysis of transmembrane proteins [14]. Through activation of Notch and NF-κB signaling pathways, PEN-2 influences cancer cell proliferation, apoptosis, and metastasis and has been proposed as a potential biomarker and therapeutic target [9].

ApoE is a secreted glycoprotein involved in lipid transport, immune regulation, and cellular proliferation. The ApoE4 isoform may contribute to gastric carcinogenesis by promoting chronic inflammation and modulating Wnt/β-catenin and PI3K/Akt signaling pathways, particularly in the context of H. Pylori-associated inflammation [15,16].

Despite accumulating evidence regarding the individual roles of these molecules, studies investigating the integrated interactions among FOXA2, angiogenic signaling, inflammatory pathways, lipid metabolism, and γ-secretase-related mechanisms remain limited. A comprehensive evaluation of these interconnected pathways may provide deeper insight into systemic regulatory networks underlying gastric cancer progression.

It should be noted that the analyzed biomarkers are not specific to gastric cancer alone and have also been implicated in neurodegenerative, inflammatory, and cardiovascular diseases. Therefore, the present study does not propose these molecules as standalone disease-specific biomarkers, but rather as components of an integrated systemic molecular network associated with gastric cancer biology.

Therefore, the aim of the present study was to investigate the circulating levels of FOXA2, Ang-1, ApoE4, PEN-2, and NF-κB in patients with gastric cancer and to evaluate their potential interactions within an integrated molecular framework reflecting transcriptional regulation, inflammation, metabolism, and angiogenesis. By simultaneously analyzing these biomarkers, this study seeks to provide a systems-level understanding of tumor–host interactions and to explore their potential utility as non-invasive indicators for disease monitoring and risk stratification.

## 2. Results

### ELISA Results of Protein Levels

**Figure 1:**
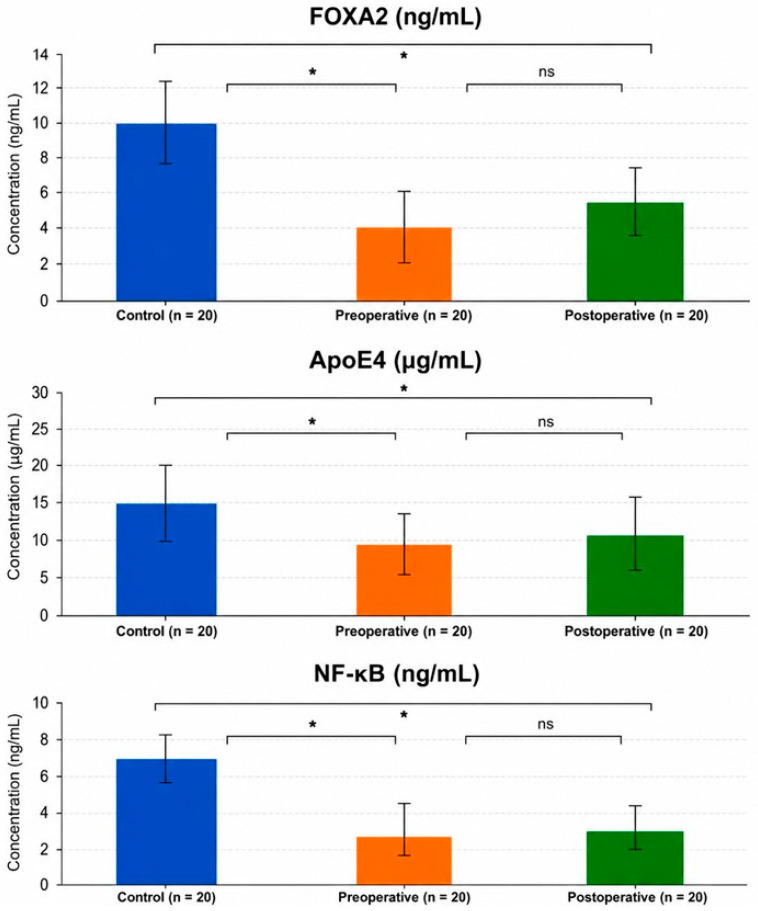
Serum FOXA2, ApoE4, and NF-κB levels measured by ELISA in healthy controls and gastric cancer patients during the preoperative and postoperative periods. FOXA2, ApoE4, and NF-κB levels were significantly lower in both preoperative and postoperative samples compared with controls (*p* < 0.05), whereas no statistically significant differences were observed between paired preoperative and postoperative measurements (ns). Data are presented as mean ± SD. * indicates *p* < 0.05; ns indicates not significant.

**Figure 2:**
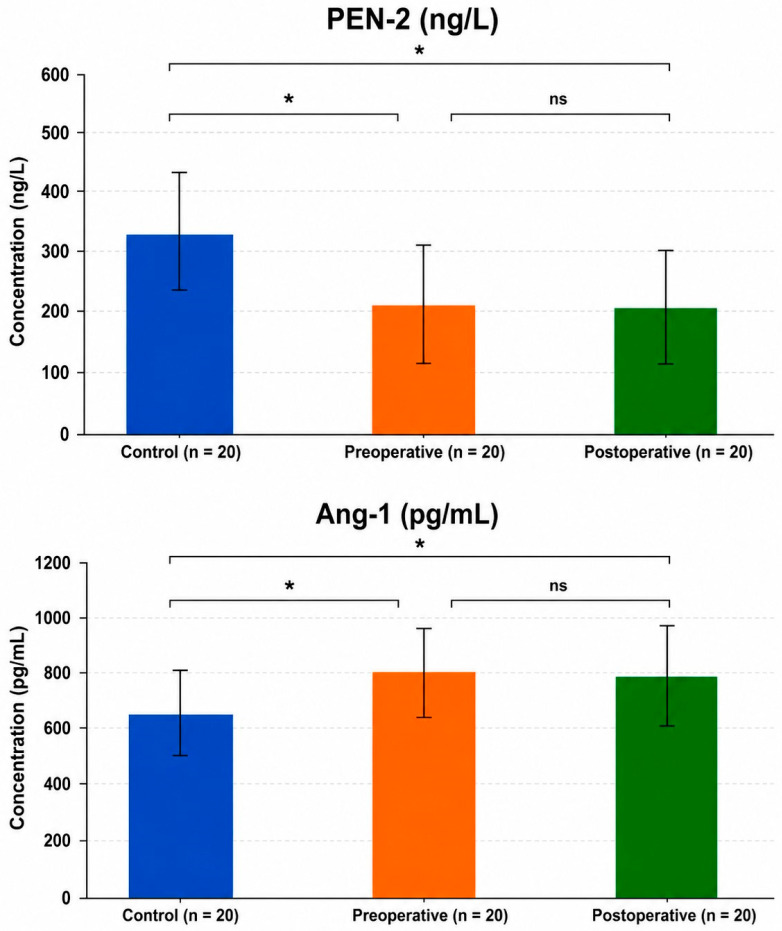
Serum PEN-2 and Ang-1 levels measured by ELISA in healthy controls and gastric cancer patients during preoperative and postoperative periods. PEN-2 levels were significantly lower, whereas Ang-1 levels were significantly higher in both preoperative and postoperative samples compared with controls (*p* < 0.05). No statistically significant differences were observed between paired preoperative and postoperative measurements (ns). Data are presented as mean ± SD. * indicates *p* < 0.05; ns indicates not significant.

Serum levels of FOXA2, Ang-1, ApoE4, PEN-2, and NF-κB were quantified by ELISA in samples obtained from healthy controls (n = 20) and from gastric cancer patients who provided paired serum samples collected during the preoperative and postoperative periods (n = 20 each). Descriptive statistics and comparative analyses are presented in Table 1. Analysis of ELISA data revealed a consistent pattern in gastric cancer patients. Specifically, serum levels of FOXA2, ApoE4, NF-κB, and PEN-2 were significantly lower in both preoperative and postoperative samples compared with the control group (*p* < 0.05) (Figure 1), whereas Ang-1 levels were significantly higher in the patient samples (*p* < 0.05) (Figure 2). Comparative analysis between the preoperative and postoperative samples demonstrated similar protein expression profiles. No statistically significant differences were detected between these paired measurements for any of the analyzed proteins, including FOXA2, Ang-1, ApoE4, PEN-2, and NF-κB (*p* > 0.05), indicating comparable serum levels before and after surgical intervention within the studied time frame.

**Figure 3:**
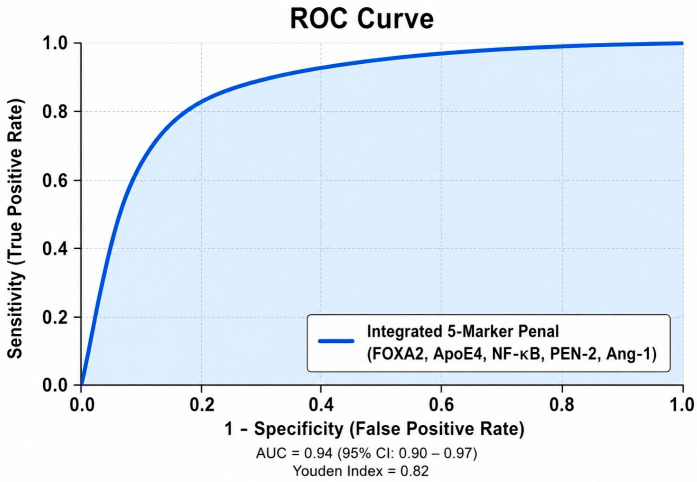
Receiver operating characteristic (ROC) curve analysis of the integrated 5-marker panel consisting of FOXA2, ApoE4, NF-κB, PEN-2, and Ang-1 for distinguishing gastric cancer patients from healthy controls. The combined biomarker panel demonstrated high diagnostic performance with an AUC of 0.94 (95% CI: 0.90–0.97). The optimal cutoff value was determined using Youden’s index (0.82).

The diagnostic potential of the integrated five-marker panel (FOXA2-NF-κB–PEN-2–ApoE4–Ang-1) was evaluated to determine its ability to distinguish gastric cancer patients from healthy controls. As illustrated in Figure 3, the combination of the sharp declines in transcriptional and inflammatory markers (FOXA2, NF-κB, PEN-2, and ApoE4) and the simultaneous increase in the angiogenic factor Ang-1 provides a highly specific non-invasive biomarker profile. The coordinated dysregulation of these markers reflects the complex tumor–host interactions and systemic biological imbalance associated with disease progression.

ROC curve analysis demonstrated that the integrated five-marker panel exhibited high diagnostic performance in distinguishing gastric cancer patients from healthy controls, with an AUC value of 0.94.

## 3. Discussion

In this study, the biomarkers were evaluated not as independent parameters but within the framework of an integrated molecular network representing systemic biological interactions. The present study demonstrates that gastric cancer is characterized by coordinated systemic alterations across transcriptional, inflammatory, metabolic, and angiogenic pathways, reflected by significant changes in circulating FOXA2, NF-κB, PEN-2, ApoE4, and Ang-1 levels. Rather than representing isolated biomarker fluctuations, these findings support the concept of an integrated molecular network shaped by dynamic tumor–host interactions.

Gastric cancer progression is driven by complex interactions among transcriptional regulation, inflammation, metabolism, and vascular remodeling; however, systemic integration of these pathways remains insufficiently understood [4,17]. FOXA2, a pioneer transcription factor regulating chromatin accessibility and epithelial differentiation, emerged as a central component of the observed molecular alterations [7,18]. Reduced circulating FOXA2 levels may reflect disruption of transcriptional programs required for gastric epithelial homeostasis. Given its role in suppressing epithelial–mesenchymal transition (EMT), decreased FOXA2 expression may indicate loss of differentiation control and increased cellular plasticity, thereby facilitating tumor progression. Furthermore, FOXA2 downregulation may influence downstream metabolic and inflammatory signaling pathways, supporting transcriptional dysregulation as an early systemic feature of gastric carcinogenesis.

NF-κB is a key mediator of inflammation-driven carcinogenesis, particularly in Helicobacter pylori-associated gastric cancer [9,19]. Although increased NF-κB activity has been demonstrated in tumor tissues, reduced circulating NF-κB levels were identified in this study. This discrepancy likely reflects compartment-specific regulation between tumor microenvironments and systemic circulation. NF-κB signaling may remain locally activated within tumor tissues while circulating levels decline due to molecular sequestration or altered immune regulation. Reduced systemic NF-κB may also indicate immune exhaustion or impaired inflammatory responsiveness, emphasizing the importance of distinguishing tissue-specific signaling activity from circulating biomarker dynamics.

In contrast to other markers, Ang-1 levels were significantly elevated in gastric cancer patients. Ang-1 promotes vascular stabilization through Tie2 receptor activation and supports endothelial survival [20,21,22]. Increased Ang-1 may represent a compensatory mechanism preserving vascular integrity under tumor-induced inflammatory stress or may facilitate tumor adaptation by promoting vascular maturation and efficient nutrient delivery without excessive vascular permeability. These findings suggest that angiogenic regulation in gastric cancer involves vascular stabilization mechanisms in addition to classical pro-angiogenic activation.

ApoE4 reduction further highlights metabolic reprogramming as a hallmark of gastric cancer progression. Cancer cells frequently alter lipid metabolism to sustain rapid proliferation, and decreased circulating ApoE4 may reflect enhanced lipid utilization or altered hepatic synthesis [23,24]. Considering ApoE4’s involvement in PI3K/Akt and Wnt/β-catenin signaling pathways, its reduction may contribute to dysregulated immune responses and chronic inflammatory conditions that promote tumor development, reinforcing the close relationship between metabolic and inflammatory processes in gastric carcinogenesis.

PEN-2, a critical component of the γ-secretase complex regulating Notch signaling, was also significantly reduced. Altered PEN-2 levels may indicate disrupted γ-secretase activity and impaired Notch–NF-κB signaling crosstalk [25,26,27,28,29,30]. Rather than uniform pathway suppression, decreased circulating PEN-2 may reflect intracellular retention or redistribution of signaling molecules during tumor progression. The parallel decline in PEN-2 and NF-κB supports coordinated regulation between these pathways.

Importantly, ROC curve analysis demonstrated that the integrated five-marker panel exhibited superior discriminative performance compared with individual biomarkers. This finding emphasizes that gastric cancer biology is better captured through multi-pathway molecular profiling rather than single-marker assessment. The combined biomarker signature likely reflects a systems-level biological imbalance integrating transcriptional, inflammatory, metabolic, and angiogenic processes.

Although the analyzed biomarkers have previously been associated with several pathological conditions, including neurodegenerative and cardiovascular diseases, the present study evaluated these molecules within the context of an integrated gastric cancer-associated molecular network rather than as isolated disease-specific markers. The combined biomarker profile may therefore reflect systemic tumor-associated biological dysregulation rather than individual disease specificity.

The absence of significant differences between preoperative and postoperative (30th day) measurements indicates that systemic molecular alterations persist even after surgical removal of the primary tumor. When considered collectively, these biomarkers appear to form an integrated molecular network reflecting complex tumor–host interactions and systemic biological dysregulation, highlighting their potential utility as a non-invasive multi-biomarker profile for disease monitoring and risk stratification.

From a clinical perspective, the identification of a non-invasive serum-based multi-marker panel may improve diagnostic stratification and disease monitoring strategies. Such an approach could complement existing diagnostic methods and potentially reduce dependence on invasive procedures if validated in larger cohorts.

Several limitations should be acknowledged, including the relatively small sample size, lack of tissue-level validation, evaluation at a single postoperative time point, absence of RNA/protein expression analyses in tumor tissues, and limited characterization of potential comorbid conditions associated with these biomarkers. Furthermore, although the analyzed biomarkers have been implicated in several neurodegenerative and cardiovascular diseases, the present study focused specifically on their integrated systemic alterations in gastric cancer rather than their disease-specific roles. Future studies involving larger and clinically well-characterized cohorts, together with integrated serum and tumor tissue RNA/protein analyses, are required to validate the specificity and translational applicability of these findings. In addition, the present findings should be interpreted within the limitations of the study and require confirmation in larger independent cohorts using complementary experimental and computational approaches.

Overall, the present findings support a systems biology model in which gastric cancer progression is reflected by coordinated circulating molecular alterations rather than single-pathway dysregulation, highlighting the value of integrated biomarker profiling for understanding disease biology and improving translational applications. These findings suggest that integrated serum biomarker profiling may support early risk stratification and longitudinal monitoring in gastric cancer management.

## 4. Materials and Methods

### 4.1. Study Design

The study was conducted in accordance with the Declaration of Helsinki and approved by the Ethics Committee of Manisa Celal Bayar University (Approval No: 20.478.486/2266). The study population consisted of 20 patients with gastric cancer who provided paired serum samples collected during the preoperative period and on the 30th postoperative day, together with 20 age-matched healthy controls. The patient group included individuals admitted to the General Surgery Clinic of Health Sciences University Dr. Abdurrahman Yurtaslan Oncology Hospital and scheduled for surgery following a diagnosis of gastric cancer.

Patients aged ≥18 years with no additional pathological conditions or chronic diseases including diabetes mellitus, hypertension, hormone replacement therapy, treatment with thyroxine derivatives, or concomitant active infectious or chronic inflammatory diseases were eligible for inclusion. Individuals with known neurodegenerative disorders, including Alzheimer’s disease and Parkinson’s disease, as well as severe cardiovascular diseases such as atherosclerosis, were excluded from the study. Written informed consent was obtained from all participants who met the inclusion criteria. Patients diagnosed with adenocarcinoma based on pathological evaluation of endoscopic biopsy specimens, who underwent preoperative staging laparoscopy and were classified as Stage 3–4, were included in the study group. None of the patients received perioperative chemotherapy or radiotherapy prior to sample collection.

For each patient in the gastric cancer group, approximately 2 mL of peripheral blood was collected as part of the study during routine blood sampling performed preoperatively and again on the 30th postoperative day. The control group (n = 20) consisted of healthy individuals within the same age range as the patient group and without any known medical conditions. Written informed consent was obtained from all participants, including the control subjects. Collected blood samples were centrifuged to separate serum and stored at −80 °C until analysis.

### 4.2. Determination of Protein Levels by ELISA

Serum levels of FOXA2, Ang-1, PEN-2, ApoE4 and NF-κB were measured using enzyme-linked immunosorbent assay (ELISA). The following commercially available sandwich ELISA kits were used: FOXA2 (BT Lab, Cat. No. E7220Hu, Shanghai, China), Ang-1 (Elabscience, Cat. No. E-EL-H6119, Wuhan, China), PEN-2 (BT Lab, Cat. No. E0980Hu, Shanghai, China), ApoE4 (Elabscience, Cat. No. E-EL-H0470, Wuhan, China), and NF-κB (Elabscience, Cat. No. E-EL-H1388, Wuhan, China). Microplates precoated with specific antibodies for each marker were incubated with serum samples, followed by the addition of detection antibodies. After color development using substrate solutions, absorbance was measured spectrophotometrically at a wavelength of 450 nm ± 2 nm.

### 4.3. Statistical Analysis

Statistical analyses were performed using SPSS software version 23.0 (SPSS Inc., Chicago, IL, USA). Data are presented as mean ± standard deviation (SD). Group comparisons between healthy controls and gastric cancer patients were initially performed using the Kruskal–Wallis test, followed by Mann–Whitney U test for pairwise comparisons when appropriate. Comparisons between preoperative and postoperative samples obtained from the same patients were performed using the Wilcoxon signed-rank test. A *p*-value of <0.05 was considered statistically significant. Non-parametric statistical analyses were preferred due to the non-normal distribution of the data.

Receiver operating characteristic (ROC) curve analysis was performed to evaluate the diagnostic performance of serum biomarkers in distinguishing gastric cancer patients from healthy controls. ROC curves were generated by plotting sensitivity (true positive rate) against 1 − specificity (false positive rate) across varying threshold values. The area under the curve (AUC) was calculated as a measure of overall diagnostic accuracy. An AUC value of 0.5 indicated no discriminative ability, whereas values approaching 1.0 represented optimal diagnostic performance. ROC analyses and graphical visualizations were performed using computational methods implemented in Python (version 3.10; Python Software Foundation, Wilmington, DE, USA). 

## 5. Conclusions

This study identifies a coordinated circulating biomarker network linking transcriptional, inflammatory, metabolic, and angiogenic pathways in gastric cancer. Rather than representing isolated disease-specific markers, the integrated biomarker profile reflects systemic tumor-associated molecular alterations and provides a systems-level perspective on gastric cancer biology. The findings highlight the potential clinical utility of non-invasive serum-based multi-marker profiling for disease monitoring and risk stratification. Nevertheless, further studies involving larger and clinically well-characterized cohorts, together with integrated tissue-level validation, are required to confirm the specificity and translational applicability of these findings.

## Figures and Tables

**Table 1:** Serum levels of PEN-2, Ang-1, FOXA2, ApoE4, and NF-κB in healthy controls and gastric cancer patients during the preoperative and postoperative periods. Data are presented as mean ± SD. Significant differences were observed between controls and patient groups (*p* < 0.05), whereas no statistically significant differences were detected between paired preoperative and postoperative samples (ns).

Group	PEN-2 (ng/L)	Ang-1 (pg/mL)	FOXA2 (ng/mL)	ApoE4 (µg/mL)	NF-κB (ng/mL)
**Control (n = 20)**	330.3 ± 90.8	671.0 ± 106.8	10.2 ± 1.7	15.0 ± 4.0	7.19 ± 0.90
**Preoperative (n = 20)**	210.1 ± 89.8	798.0 ± 105.7	4.26 ± 1.70	9.62 ± 2.90	2.82 ± 0.80
**Postoperative (n = 20)**	208.3 ± 81.0	799.1 ± 189.0	5.46 ± 1.60	10.66 ± 2.90	3.00 ± 0.60

## Data Availability

The original contributions presented in this study are included in the article. Further inquiries can be directed to the corresponding author.

## Abbreviations

FOXA2	Forkhead Box A2
NF-κB	Nuclear Factor kappa B
Ang-1	Angiopoietin-1
PEN-2	Presenilin Enhancer-2
ApoE4	Apolipoprotein E4
ROC	Receiver Operating Characteristic
ELISA	Enzyme-Linked Immunosorbent Assay

## References

[1] Zhang T., Zhang Y., Leng X. (2024). Global, regional, and national trends in gastric cancer burden: 1990–2021 and projections to 2040. Front. Oncol..

[2] Agboola O. (1994). Adjuvant treatment in gastric cancer. Cancer Treat. Rev..

[3] Cunningham D., Allum W.H., Stenning S.P., Thompson J.N., Van de Velde C.J., Nicolson M., Scarffe J.H., Lofts F.J., Falk S.J., Iveson T.J. (2006). Perioperative chemotherapy versus surgery alone for resectable gastroesophageal cancer. N. Engl. J. Med..

[4] Zhu C.P., Wang J., Shi B., Hu P.F., Ning B.F., Zhang Q., Chen F., Chen W.S., Zhang X., Xie W.F. (2015). The transcription factor FOXA2 suppresses gastric tumorigenesis in vitro and in vivo. Dig. Dis. Sci..

[5] Tuteja G., Kaestner K.H. (2007). SnapShot: Forkhead transcription factors I. Cell.

[6] Lehmann O.J., Sowden J.C., Carlsson P., Jordan T., Bhattacharya S.S. (2003). Fox’s in development and disease. Trends Genet..

[7] Liu N., Wang A., Xue M., Zhu X., Liu Y., Chen M. (2024). FOXA1 and FOXA2: The regulatory mechanisms and therapeutic implications in cancer. Cell Death Discov..

[8] Kawasaki K., Salehi S., Zhan Y.A., Chen K., Lee J.H., Salataj E., Zhong H., Manoj P., Kinyua D., Mello B.P. (2025). FOXA2 promotes metastatic competence in small cell lung cancer. Nat. Commun..

[9] Sokolova O., Naumann M. (2017). NF-κB signaling in gastric cancer. Toxins.

[10] Gochman E., Mahajna J., Reznick A.Z. (2011). NF-κB activation by peroxynitrite through IκBα-dependent phosphorylation versus nitration in colon cancer cells. Anticancer Res..

[11] Wang Z., Zhao Y., An Z., Li W. (2019). Molecular links between angiogenesis and neuroendocrine phenotypes in prostate cancer progression. Front. Oncol..

[12] Akwii R.G., Sajib M.S., Zahra F.T., Mikelis C.M. (2019). Role of angiopoietin-2 in vascular physiology and pathophysiology. Cells.

[13] Gu G., Zhu B., Ren J., Song X., Fan B., Ding H., Shang J., Wu H., Li J., Wang H. (2023). Ang-(1-7)/MasR axis promotes functional recovery after spinal cord injury by regulating microglia/macrophage polarization. Cell Biosci..

[14] Serneels L., Bammens L., Zwijsen A., Tolia A., Chávez-Gutiérrez L., De Strooper B. (2023). Functional and topological analysis of PSENEN, the fourth subunit of the γ-secretase complex. J. Biol. Chem..

[15] Miao G., Zhuo D., Han X., Yao W., Liu C., Liu H., Cao H., Sun Y., Chen Z., Feng T. (2023). From degenerative disease to malignant tumors: Insight into the function of ApoE. Biomed. Pharmacother..

[16] Sakashita K., Tanaka F., Zhang X., Mimori K., Kamohara Y., Inoue H., Sawada T., Hirakawa K., Mori M. (2008). Clinical significance of ApoE expression in human gastric cancer. Oncol. Rep..

[17] Maddineni G., Xie J.J., Brahmbhatt B., Mutha P. (2022). Diet and carcinogenesis of gastric cancer. Curr. Opin. Gastroenterol..

[18] Orstad G., Fort G., Parnell T.J., Jones A., Stubben C., Lohman B., Gillis K.L., Orellana W., Tariq R., Klingbeil O. (2022). FoxA1 and FoxA2 control growth and cellular identity in NKX2-1-positive lung adenocarcinoma. Dev. Cell.

[19] Chaithongyot S., Jantaree P., Sokolova O., Naumann M. (2021). NF-κB in gastric cancer development and therapy. Biomedicines.

[20] Khan K.A., Wu F.T., Cruz-Munoz W., Kerbel R.S. (2021). Ang2 inhibitors and Tie2 activators: Potential therapeutics in perioperative treatment of early stage cancer. EMBO Mol. Med..

[21] Liu N., Liu M., Fu S., Wang J., Tang H., Isah A.D., Chen D., Wang X. (2022). Ang2-targeted combination therapy for cancer treatment: Preclinical rationale and challenges. Front. Immunol..

[22] Sun X.D., Liu X.E., Wu J.M., Cai X.J., Mou Y.P., Li J.D. (2004). Expression and significance of angiopoietin-2 in gastric cancer. World J. Gastroenterol..

[23] Chen J., Zhu H., Chen S., Mi H. (2024). Apolipoprotein E is a potential biomarker for predicting cancer prognosis and is correlated with immune infiltration. Onco Targets Ther..

[24] Shi X., Xu J., Wang J., Cui M., Gao Y., Niu H., Jin H. (2015). Expression analysis of apolipoprotein E and its associated genes in gastric cancer. Oncol. Lett..

[25] Song C., Zhang J., Xu C., Gao M., Li N., Geng Q. (2023). The critical role of γ-secretase and its inhibitors in cancer and cancer therapeutics. Int. J. Biol. Sci..

[26] Yang Z., Wen D., Ye Y., Chen K., Qiu Z., Liu X. (2023). Pan-cancer analysis highlights the role of PSENEN (PEN-2) in tumor progression. J. Holist. Integr. Pharm..

[27] Huang C., Chen K., Zhu S., Yang X., Hou J., Gu X. (2024). PSENEN influences the progression of renal clear cell carcinoma by regulating the immune microenvironment and oxidative phosphorylation. PeerJ.

[28] Liu X., Yan C., Chang C., Meng F., Shen W., Wang S., Zhang Y. (2023). FOXA2 suppression by TRIM36 exerts anti-tumor role in colorectal cancer via inducing NRF2/GPX4-regulated ferroptosis. Adv. Sci..

[29] Perks C.M., Barker R.M., Alhadrami M., Alkahtani O., Gill R., Grishaw M., Harland A.J., Henley P., Li H., O’Sullivan E. (2025). Curious dichotomies of apolipoprotein E function in Alzheimer’s disease and cancer: One explanatory mechanism of inverse disease associations?. Genes.

[30] Wang Z., Chen H., Sun L., Wang X., Xu Y., Tian S., Liu X. (2024). Uncovering APOD/APOE family members as biomarkers in gastric cancer: Transcriptomic profiling and clinical correlation. Comput. Struct. Biotechnol. J..

